# Supplementary material for: Investigating brain alterations in the Dp1Tyb mouse model of Down syndrome
Source: Neurobiol Dis. Author manuscript; Available in PMC 2024 Feb 5. (PMC7615598; doi:10.1016/j.nbd.2023.106336)
Supplement: Supplementary Fig. 1 [file EMS193704-supplement-Supplementary_Fig__1.docx]

# SUPPLEMENTARY METHODS

## TENSOR-BASED MORPHOMETRY AND ATLAS-BASED ANALYSIS

### *In vivo* analysis

A study-specific template was created from the T1-weighted images using the Advanced Normalization Tools (ANTs) software (antsMultivariateTemplateConstruction2.sh). All subjects were registered to this template via sequential rigid, affine, and nonlinear SyN transforms (antsRegistration). Then, the geometric Jacobian determinant maps of the resultant deformation fields were computed and log-transformed (CreateJacobianDeterminantImage) and T1 maps were warped to the study template using the same transformations (antsApplyTransforms) for nonparametric permutation testing using FSL randomise, performing 5000 permutations, threshold-free cluster enhancement, and family-wise error correction.

The study template was masked using the artsBrainExtraction algorithm (MacNicol et al., 2021) to perform atlas-based brain extraction by registering the template to the MouseIn in vivo MP2RAGE templates of the C57Bl6/J mouse brain (<https://doi.org/10.17605/OSF.IO/SZYQE>). The template brain mask was warped to each subject space using the inverse of the transforms from the subject-to-template registrations. MATLAB was used to calculate the volume (V) and surface area (A) of each subject’s brain mask (regionprops3), and the sphericity (Ψ) was calculated using the formula $\Psi=\pi^{\frac{1}{3}}\left( 6V \right)^{\frac{2}{3}}/A$.

For atlas-based regional analysis, the masked study template was registered to the Allen mouse brain common coordinate framework (CCFv3) (Wang et al., 2020). The original CCFv3 brain parcellation was condensed into 72 regions of interest (ROI) (Supplementary Table 1), and these ROIs were warped to the study template. The different ROI volumes were calculated by summing the Jacobian determinant values within each ROI. The mean T1 value of each ROI was also computed. To calculate whole cerebellum volumes, a cerebellum mask was created by merging the arbor vitae, cerebellar cortex, cerebellar nuclei, and flocculus/paraflocculus ROIs. This cerebellum mask was warped to each subject space, and the volumes of the subject cerebellum masks calculated as described for the total brain mask.

### *Ex vivo* analysis

Tensor-based morphometry and atlas-based regional analysis were performed on the ex vivo images using a similar pipeline. The only difference was that the ex vivo images were first masked using artsBrainExtraction and the DSURQE template (Dorr et al., 2008) to remove the varying amounts of residual soft tissue on top of the skulls. A study template was created from the masked images. Total brain volume, surface area, and sphericity were calculated from the subject masks.

# SUPPLEMENTARY DATA

## SUPPLEMENTARY TABLES

### Supplementary Table 1. Regions of interest in the modified Allen Mouse Brain Atlas.

| 1 | amygdala |
| --- | --- |
| 2 | arbor vitae |
| 3 | auditory cortex |
| 4 | bed nuclei |
| 5 | cerebellar cortex |
| 6 | cerebellar nuclei |
| 7 | cerebellar white matter |
| 8 | cerebral aqueduct |
| 9 | choroid plexus |
| 10 | cingulate cortex |
| 11 | claustrum |
| 12 | corpus callosum |
| 13 | cortical subplate |
| 14 | diagonal band |
| 15 | dorsal peduncular area |
| 16 | entorhinal cortex |
| 17 | fiber tracts |
| 18 | fimbria fornix |
| 19 | Flocculus/paraflocculus |
| 20 | fourth ventricle |
| 21 | frontal cortex |
| 22 | gustatory and visceral cortex |
| 23 | habenula |
| 24 | hippocampus dorsal |
| 25 | hippocampus ventral |
| 26 | hypothalamus |
| 27 | inferior colliculus |
| 28 | infralimbic cortex |
| 29 | insular cortex |
| 30 | internal capsule |
| 31 | lateral geniculate |
| 32 | lateral ventricle |
| 33 | medial geniculate |
| 34 | medulla |
| 35 | midbrain |
| 36 | midbrain white matter |
| 37 | motor cortex |
| 38 | nodulus |
| 39 | nucleus accumbens |
| 40 | nucleus of posterior commussure |
| 41 | olfactory cortex |
| 42 | olfactory tracts |
| 43 | optic tract |
| 44 | orbital cortex |
| 45 | pallidum |
| 46 | parietal cortex |
| 47 | pedunculopontine nucleus |
| 48 | periaqueductal gray |
| 49 | perirhinal cortex |
| 50 | piriform cortex |
| 51 | pons |
| 52 | postrhinal cortex |
| 53 | prelimbic cortex |
| 54 | pretectal |
| 55 | raphe |
| 56 | red nucleus |
| 57 | reticular nucleus |
| 58 | retrosplenial cortex |
| 59 | secondary sensory cortex |
| 60 | sensory cortex |
| 61 | septum |
| 62 | stria terminalis tracts |
| 63 | striatum |
| 64 | subiculum |
| 65 | substantia nigra |
| 66 | superior colliculus |
| 67 | taenia tecta |
| 68 | thalamus |
| 69 | third ventricle |
| 70 | visual cortex |
| 71 | white matter |
| 72 | zona incerta |

### Supplementary Table 2. Details of antibodies used to perform free-floating immunofluorescence. In grey, antibodies used for GFAP/NeuN IF and in white, those used for Iba1/SV2A IF.

|  | **Antibody** | **Supplier** | **Catalog number** | **Dilution** |
| --- | --- | --- | --- | --- |
| Primary antibodies | chicken anti-NeuN | Synaptic systems | #266006 | 1:200 |
|  | rabbit anti-GFAP | DAKO | #Z-0334 | 1:1000 |
|  | goat anti-Iba1 | Abcam | #ab5076 | 1:1000 |
|  | rabbit anti-SV2A | Abcam | #ab32942 | 5µg/ml |
| Secondary antibodies | Alexa Fluor 488 donkey anti-chicken IgY | Jackson Immuno | #703-545-155 | 1:600 |
|  | Alexa Fluor 568 donkey anti-rabbit | Thermo Fisher | #A10042 | 1:600 |
|  | Alexa fluor 488 donkey anti-goat | Jackson Immuno | #705-605-147 | 1:3000 |
|  | Alexa fluor 568 donkey anti-rabbit | Thermo Fisher | #A10042 | 1:500 |

|  | *In vivo* | | *Ex vivo* | |
| --- | --- | --- | --- | --- |
|  | WT  (7m, 6f) | Dp1Tyb  (4m, 5f) | WT  (8m, 6f) | Dp1Tyb  (5m, 3f) |
| WB (mm^3^) m  WB (mm^3^) f | 470.7 ± 5.53  475.9 ± 5.97 | 445.3 ± 7.31  480.7 ± 6.54^#^ | 506.3 ± 2.6  504.7 ± 4.53 | 485.9 ± 8.21 **  491.2 ± 5.90 |
| CB (mm^3^) m  CB (mm^3^) f | 49.1 ± 0.56  49.8 ± 0.61 | 45.2 ± 0.74 **  49.8 ± 0.66^#^ | 52.5 ± 0.82  51.1 ± 0.94 | 46.9 ± 1.03 **  48.8 ± 1.33 |
| CB % of WB m  CB % of WB f | 10.5 ± 0.12  10.2 ± 0.15 | 10.3 ± 0.15  10.6 ± 0.17 | 10.4 ± 0.17  9.8 ± 0.19 | 9.8 ± 0.23 *  10.2 ± 0.21 |

### Supplementary Table 3. Comparison of in vivo and ex vivo whole brain (WB) and cerebellum (CB; absolute and relative) volume measurements. Data are presented as mean ± SEM (m, males; f, females). For the in vivo measures, WB volumes showed a significant sex x genotype interaction (F_1,18_=5.59, p=0.030) as well as a significant effect of sex (F_1,18_=10.14, p=0.005), with male Dp1Tyb mice smaller than female (^#^p=0.026), but no effect of genotype. In vivo measured CB volumes showed significant effect of genotype (**F_1,18_=9.20, p=0.007), sex (F_1,18_=16.55, p<0.001) and genotype x sex interaction (F_1,18_=8.62, p=0.009), with male Dp1Tyb mice smaller than female (^#^p=0.001). There were no differences in in vivo CB normalised to WB (CB % of WB). Ex vivo measured WB, absolute and normalised CB volumes, all showed significant effect of genotype (**F_1,18_=8.30, p=0.010, **F_1,18_=14.06, p=0.002, *F_1,18_=5.36, p=0.033, respectively) with no effect of sex nor interaction.

### Supplementary Table 4. Regions with significantly different *in vivo* volume in Dp1Tyb mice compared to WT. Dp1Tyb mice (n = 9) had a significantly different volume in 26 out of 73 examined ROIs, compared to WT (n= 13). These group differences have been evaluated with a two-way ANOVA (genotype × sex), using the false discovery rate (FDR) to correct for multiple comparisons (p < 0.007, Q = 5%), effect size also shown (Cohen’s d). Regions that are larger in Dp1Tyb brains compared to WT are indicated in red.

| REGION | FUNCTION | Difference | F | p | Cohen’s d |
| --- | --- | --- | --- | --- | --- |
| Orbital cortex | Decision making & executive process | WT > Dp1Tyb | F_1,18_ = 12.77 | 0.002 | 1.46 |
| Prelimbic cortex |  | WT > Dp1Tyb | F_1,18_ = 20.14 | < 0.001 | 1.84 |
| Infralimbic cortex |  | WT > Dp1Tyb | F_1,18_ = 30.47 | < 0.001 | 2.36 |
| Retrosplenial cortex | Working memory & spatial memory tasks | WT > Dp1Tyb | F_1,18_ = 32.48 | < 0.001 | 2.47 |
| Dorsal hippocampus |  | WT > Dp1Tyb | F_1,18_ = 27.09 | < 0.001 | 2.19 |
| Motor cortex | Processing of sensory and sensorimotor stimuli | WT > Dp1Tyb | F_1,18_ = 15.09 | 0.001 | 1.63 |
| Sensory Cortex |  | WT > Dp1Tyb | F_1,18_ = 13.54 | 0.002 | 1.45 |
| Auditory cortex |  | WT > Dp1Tyb | F_1,18_ = 9.09 | 0.007 | 0.93 |
| Olfactory cortex |  | WT > Dp1Tyb | F_1,18_ = 17.88 | 0.001 | 1.77 |
| Olfactory tracts |  | WT > Dp1Tyb | F_1,18_ = 21.62 | < 0.001 | 1.99 |
| Piriform cortex |  | WT > Dp1Tyb | F_1,18_ = 10.77 | 0.004 | 1.3 |
| Claustrum |  | WT > Dp1Tyb | F_1,18_ = 12.33 | 0.002 | 1.09 |
| Parietal cortex |  | WT > Dp1Tyb | F_1,18_ = 12.54 | 0.002 | 1.52 |
| Habenula | Stress & Anxiety | WT > Dp1Tyb | F_1,18_ = 12.24 | 0.003 | 1.7 |
| Dorsal peduncular area |  | WT > Dp1Tyb | F_1,18_ = 16.28 | 0.001 | 1.69 |
| Thalamus | Emotional response | WT > Dp1Tyb | F_1,18_ = 11.98 | 0.003 | 1.15 |
| Insular cortex |  | WT > Dp1Tyb | F_1,18_ = 17.64 | 0.001 | 1.67 |
| Cingulate cortex |  | WT > Dp1Tyb | F_1,18_ = 29.35 | < 0.001 | 2.19 |
| Amygdala |  | WT > Dp1Tyb | F_1,18_ = 10.01 | 0.005 | 1.33 |
| Septum |  | WT < Dp1Tyb | F_1,18_ = 9.53 | 0.006 | -1.39 |
| Fourth ventricle | Cerebrospinal fluid production | WT > Dp1Tyb | F_1,18_= 9.25 | 0.007 | 1.36 |
| Internal capsule | Cortex-brainstem communication | WT > Dp1Tyb | F_1,18_ = 10.26 | 0.005 | 1.32 |
| Pons | Regulation of sleep-wake cycle & autonomic functions | WT < Dp1Tyb | F_1,18_ = 13.34 | 0.002 | -1.65 |
| Pontine reticular nucleus |  | WT < Dp1Tyb | F_1,18_ = 15.17 | 0.001 | -1.11 |
| Pedunculopontine nucleus |  | WT < Dp1Tyb | F_1,18_ = 25.56 | < 0.001 | -2.29 |
| Flocculus | Motor control – vestibulo-ocular reflex system | WT > Dp1Tyb | F_1,18_ = 24.79 | < 0.001 | 2.18 |

### Supplementary Table 5. Metabolites measured by MRS (mM, except Gln/Glu; mean ± sem). Data were analysed by two-way (genotype × sex) ANOVA and p values show the effect of genotype, * p<0.05, ** p<0.01, ***p<0.001, as well as effect size (Cohen’s d). Three metabolites (glutamine, taurine and glutamine/glutamate ratio, in red) remained significant after correction for multiple comparisons (q-value set as 0.05) controlling the false discovery rate (FDR).

| **MRS metabolites** | **WT** | **Dp1Tyb** | **Effect size (Cohen’s d)** |
| --- | --- | --- | --- |
|  | males / females | males / females |  |
| creatine (Cr) | 3.19±0.14 / 2.97±0.10 | 2.92±0.28 / 2.82±0.24 | -0.71 |
| gamma-aminobutyric acid (GABA) | 1.85±0.09 / 1.94±0.08 | 2.00±0.09 / 1.88±0.06 | 0.17 |
| glutamine (Gln) | 1.98±0.05 / 2.02±0.06 | ↑ 3.39±0.88 / 3.31±0.30 *** | 10.00 |
| glutamine/glutamate (Gln/Glu) | 0.31±0.01 / 0.32±0.01 | ↑ 0.60±0.17 / 0.56±0.07 *** | 9.43 |
| glutamate (Glu) | 6.30±0.07 / 6.32±0.13 | ↓ 5.88±0.26 / 5.97±0.22 * | -1.54 |
| glutathione (GSH) | 1.39±0.02 / 1.43±0.04 | ↓ 1.38±0.03 / 1.30±0.04 * | -0.97 |
| myo-inositol (Ins) | 3.63±0.11 / 3.91±0.10 | 3.88±0.55 / 3.46±0.31 | -0.39 |
| lactate (Lac) | 1.38±0.06 / 1.34±0.16 | ↑ 1.80±0.35 / 2.01±0.18 ** | 2.01 |
| N-acetyl-aspartate (NAA) | 5.41±0.06 / 5.44±0.06 | ↓ 5.25±0.11 / 5.17±0.12 * | -1.58 |
| phosphocholine (PCh) | 0.75±0.02 / 0.76±0.05 | 0.81±0.10 / 0.80±0.08 | 0.73 |
| taurine (Taur) | 6.91±0.20 / 6.19±0.17 | ↓ 5.33±0.31 / 5.21+0.40 *** | -2.24 |

### Supplementary Table 6. *Ex-vivo* differences in cerebellar thickness and/or volume between 3-month-old WT and Dp1Tyb mice. Cerebellar ROIs where we can observe differences in cerebellar thickness and/or volume between WT (n= 13) and Dp1Tyb (n = 9) calculated with a two-way ANOVA (genotype × sex). In red, the statistically significant differences found after multiple comparisons correction using the false discovery rate (FDR) (p < 0.0032, Q = 5%). Abbreviations: lobules of the cerebellar vermis: 3Cb (lobule 3), 4/5Cb (lobule 4/5), 7Cb (lobule 7), 8Cb (lobule 8), 9Cb (lobule 9), 10Cb (lobule 10); lobules of cerebellar hemispheres: Crus 1 (Crus 1 of the ansiform lobule), Crus 2 (Crus 2 of the ansiform lobule), PM (paramedian lobule), Cop (Copula of the pyramis), PFI (Paraflocculus), FI (Flocculus).

| REGION | MOLECULAR layer | | GRANULAR layer | |
| --- | --- | --- | --- | --- |
|  | THICKNESS | VOLUME | THICKNESS | VOLUME |
| 3Cb | p < 0.05 | F_1,18_ = 34.66,  p < 0.0001 | F_1,18_ = 8.95,  p = 0.008 | F_1,18_ = 18.21,  p = 0.0005 |
| 4/5Cb | p < 0.05 | p < 0.05 | p < 0.05 | F_1,18_ = 5.08,  p = 0.037 |
| 7Cb | p < 0.05 | F_1,18_ = 7.32,  p = 0.015 | p < 0.05 | p < 0.05 |
| 8Cb | F_1,18_ = 10.74,  p = 0.004 | F_1,18_ = 21.84,  p = 0.0002 | F_1,18_ = 10.39,  p = 0.005 | F_1,18_ = 16.69,  p = 0.0007 |
| 9Cb | p < 0.05 | F_1,18_ = 5.87,  p = 0.026 | p < 0.05 | F_1,18_ = 17.17,  p = 0.0006 |
| 10Cb | F_1,18_ = 8.41,  p = 0.01 | F_1,18_ = 8.01,  p = 0.011 | F_1,18_ = 9.65,  p = 0.006 | F_1,18_ = 15.53,  p = 0.001 |
| Crus1 | p < 0.05 | p < 0.05 | F_1,18_ = 10.41,  p = 0.005 | F_1,18_ = 7.55,  p = 0.013 |
| Crus2 | F_1,18_ = 5.32,  p = 0.033 | F_1,18_ = 6.18,  p = 0.023 | p < 0.05 | F_1,18_ = 6.44,  p = 0.021 |
| Cop | p < 0.05 | p < 0.05 | p < 0.05 | F_1,18_ = 9.24,  p = 0.007 |
| PFI | p < 0.05 | F_1,18_ = 8.62,  p = 0.008 | p < 0.05 | F_1,18_ = 16.64,  p = 0.0007 |
| FI | p < 0.05 | F_1,18_ = 14.84,  p = 0.001 | F_1,18_ = 10.54,  p = 0.005 | F_1,18_ = 13.61,  p = 0.002 |
| ALL | p < 0.05 | F_1,18_ = 7.43,  p = 0.014 | F_1,18_ = 7.18,  p = 0.015 | F_1,18_ = 11.74,  p = 0.003 |

## SUPPLEMENTARY FIGURES


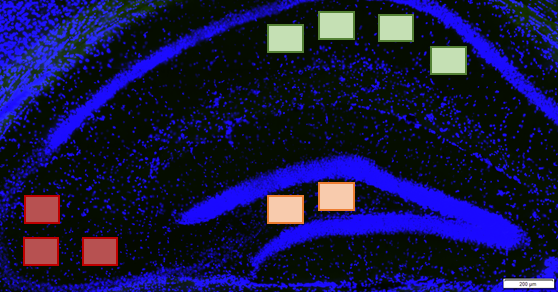


### Supplementary Figure 1. Hippocampal subregions selected for the immunofluorescence (IF) analysis. Representative hippocampal image after DAPI counterstaining of nuclei and the three ROIs used for the IF analysis: CA1 (green), CA3 (red), DG (orange). Image captured at 10x magnification with a Virtual Slide Microscope VS120 (Olympus Life Science). Scale bar: 200 µm.


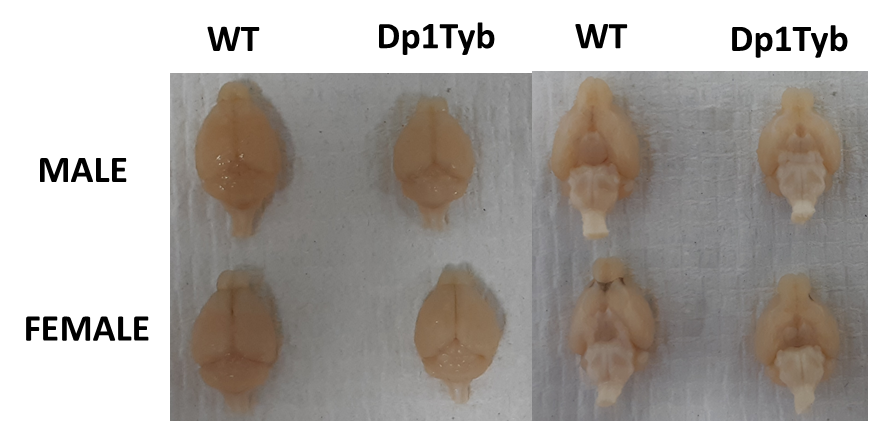


Supplementary Figure 2. Representative brains of 3-month-old WT and Dp1Tyb mice. Animals were perfused with heparinized saline and 5% PFA. Photos were taken after brain extraction, showing differences in the shape of representative WT and Dp1Tyb brains, of both sexes. The Dp1Tyb brains are smaller and rounder than the WT ones.

###
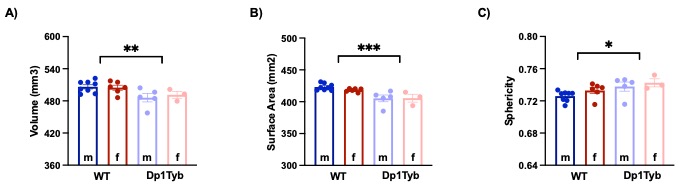


**Supplementary Figure 3. Ex vivo brain characteristics of wild-type (WT) and Dp1Tyb mice.** Quantification of A) brain volume, B) surface area, and C) sphericity of WT (males = 8, females = 6) vs Dp1Tyb (males = 5, females = 3). Bars represent the mean ± SEM. (*), (**) and (***) indicate significant differences between WT and Dp1Tyb mice (genotype effect) in the three brain characteristics analysed (F_1,18_ =8.30, p=0.01**, F_1,18_ =19.87, p<0.001*** and F_1,18_ =6.61, p=0.019*, respectively) as yielded by a two-way ANOVA (genotype × sex). m: male, f: female.


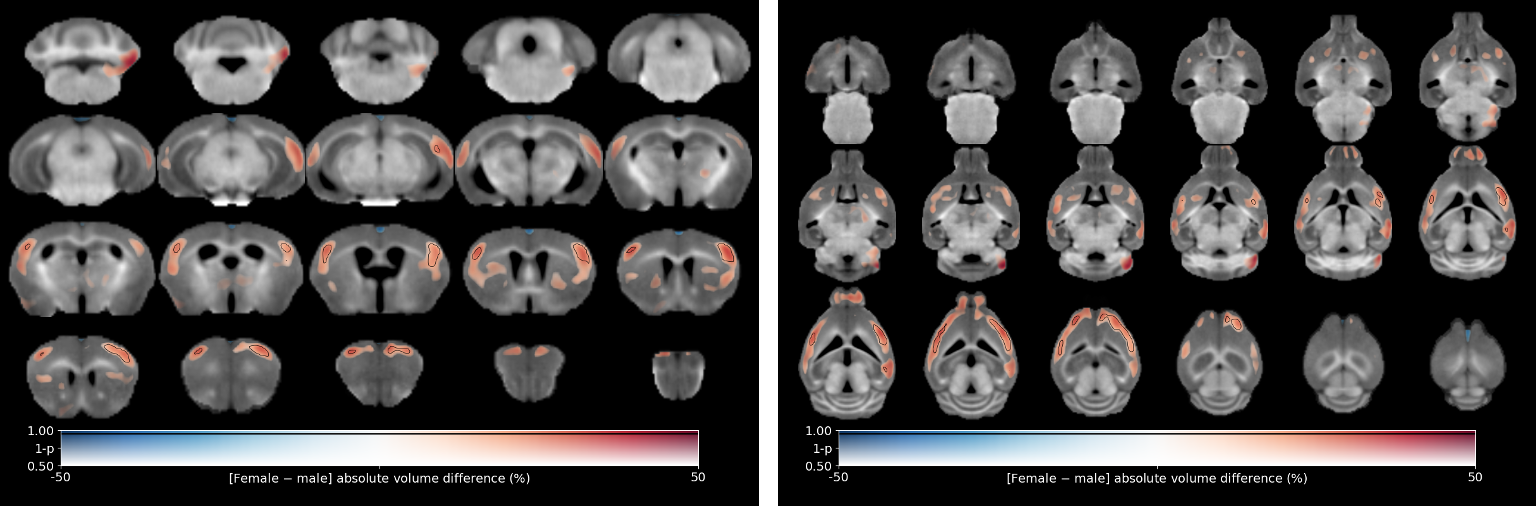


### Supplementary Figure 4. Voxel-wise differences in volume between 3-month-old male and female mice (both genotypes combined together). Map of voxel-wise differences, derived from in vivo MR images and overlaid on the T1-weighted study-specific template. The map is displayed in the coronal plane (left image, caudal-rostral) and the horizontal plane (right image, ventral-dorsal). *For reference, the coronal slices in the left column are (approximately): bregma -7.6, -4.6, -1.6, 1.4 mm (0.6 mm distance between adjacent slices), respectively.* The colour of the overlay indicates the percent volume difference (hot colours indicate increased volume in female compared to male), and the opacity of the overlay indicates the significance of the volume difference (regions where the FWE-corrected p > 0.5 are completely transparent, and regions where the FWE-corrected p = 0 completely opaque). Clusters where the FWE-corrected p < 0.05 are contoured in black.


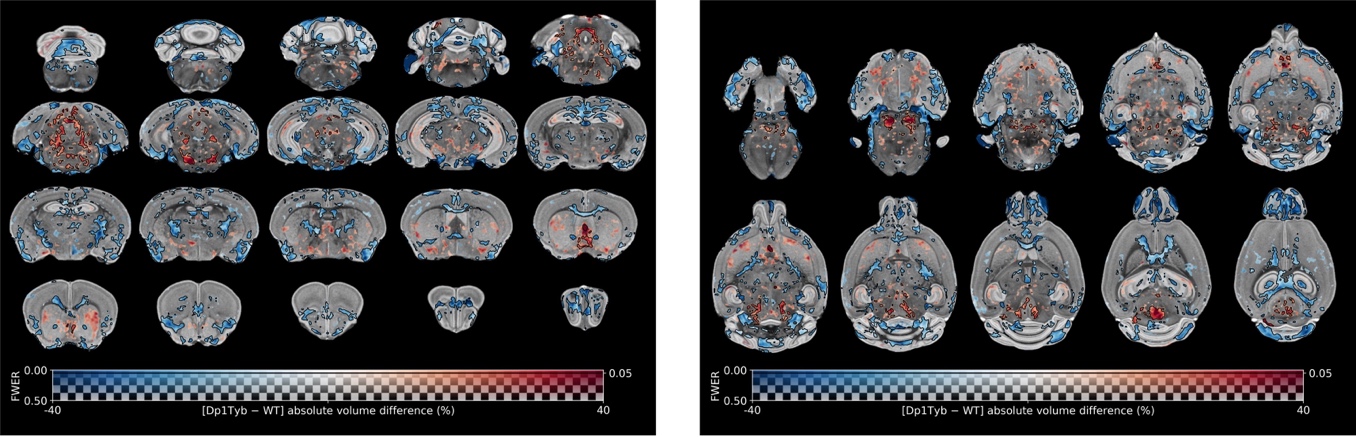


### Supplementary Figure 5. Ex-vivo differences in volume between 3-month-old WT and Dp1Tyb mice. Map of voxel-wise differences in volume, calculated from ex vivo MR images and overlaid on the Allen mouse brain template. The map is displayed in the coronal plane (left image, caudal-rostral) and the horizontal plane (right image, ventral-dorsal). For reference, the coronal slices in the left column are (approximately): bregma -7.6, -4.6, -1.6, 1.4 mm (0.6 mm distance between adjacent slices), respectively. The colour of the overlay indicates the percent volume difference (cool colours indicate reduced volume in Dp1Tyb mice, n = 9 compared to WT, n = 13), and the opacity of the overlay indicates the significance of the volume difference (regions where the FWE-corrected p > 0.5 are completely transparent, and regions where the FWE-corrected p = 0 completely opaque). Clusters where the FWE-corrected p < 0.05 are contoured in black.

# REFERENCES

Dorr, A.E., Lerch, J.P., Spring, S., Kabani, N., Henkelman, R.M., 2008. High resolution three-dimensional brain atlas using an average magnetic resonance image of 40 adult C57Bl/6J mice. Neuroimage 42, 60–69. https://doi.org/10.1016/j.neuroimage.2008.03.037

MacNicol, E., Ciric, R., Kim, E., Censo, D. Di, Cash, D., Poldrack, R.A., Esteban, O., 2021. Atlas-Based Brain Extraction Is Robust Across RAT MRI Studies, in: 2021 IEEE 18th International Symposium on Biomedical Imaging (ISBI). IEEE, pp. 312–315. https://doi.org/10.1109/ISBI48211.2021.9433884

Wang, Q., Ding, S.-L., Li, Y., Royall, J., Feng, D., Lesnar, P., Graddis, N., Naeemi, M., Facer, B., Ho, A., Dolbeare, T., Blanchard, B., Dee, N., Wakeman, W., Hirokawa, K.E., Szafer, A., Sunkin, S.M., Oh, S.W., Bernard, A., Phillips, J.W., Hawrylycz, M., Koch, C., Zeng, H., Harris, J.A., Ng, L., 2020. The Allen Mouse Brain Common Coordinate Framework: A 3D Reference Atlas. Cell 181, 936-953.e20. https://doi.org/10.1016/j.cell.2020.04.007
